# Endovascular treatment of a giant aneurysm of the proper hepatic artery: A case report

**DOI:** 10.1016/j.radcr.2025.04.130

**Published:** 2025-05-29

**Authors:** Thomas J. Vogl, Carolyn Enam Pi-Bansa, Kyriakos Oikonomou, John Bielfeldt, Hamzah Adwan

**Affiliations:** a Clinic for Radiology and Nuclear Medicine, University Hospital Frankfurt, Goethe, University, Theodor-Stern-Kai 7, 60590, Frankfurt Am Main, Germany; b Department of Vascular and Endovascular Surgery, University Hospital Frankfurt, Goethe University, Theodor-Stern-Kai 7, 60590, Frankfurt Am Main, Germany

**Keywords:** Interventional radiology, Aneurysm, Proper hepatic artery, Stent

## Abstract

Hepatic artery aneurysms (HAAs) are rare vascular abnormalities, accounting for 20% of all visceral arterial aneurysms. Nontraumatic HAAs are often asymptomatic and are typically discovered incidentally during imaging for unrelated conditions. However, they pose a significant risk of rupture, which can lead to life-threatening hemorrhaging. We present the case of a 65-year-old male with an asymptomatic saccular aneurysm of the proper hepatic artery diagnosed incidentally on CT scan. Further cross-sectional imaging confirmed the diagnosis and revealed no evidence of rupture. The patient underwent successful interventional endovascular repair using a covered stent under local anesthesia. This case emphasizes the use of minimally invasive techniques of interventional radiology in the management of complex vascular pathologies.

## Introduction

Hepatic artery aneurysms (HAAs) are rare vascular abnormalities that account for approximately 20% of visceral artery aneurysms [1]. Due to their low incidence rate, definitive risk factors are difficult to pinpoint. Studies have reported various risk factors such as, trauma, atherosclerosis and vascular disease, as well as pancreatitis, infections and connective tissue disorders, or additionally more increasingly iatrogenic causes [2,3]. Hereby, traumatic and iatrogenic aneurysms are often false aneurysms [4]. Aneurysms are most frequently located in the common hepatic artery, followed by the proper hepatic artery and right hepatic artery [1].

Nontraumatic HAAs are rare and often remain asymptomatic until complications such as rupture occur. This makes early diagnosis critical, as rupture can lead to life-threatening hemorrhage or sepsis with high mortality rates [1,3,5]. The clinical presentation of HAAs may range from nonspecific abdominal pain and obstructive jaundice to acute hemodynamic compromise due to rupture [1,2].

Unruptured, nontraumatic HAAs are increasingly being discovered incidentally during imaging studies conducted for other purposes, due to the widespread use of advanced imaging modalities [2,6]. HAAs are most often diagnosed incidentally via computed tomography (CT) or ultrasound; however, to definitively localize and anatomically characterize these aneurysms, an additional angiogram using CT or magnetic resonance imaging (MRI) should be performed [2,3].

Management strategies for HAAs range from conservative monitoring to invasive interventions, depending on various factors such as aneurysm size, symptomatic presentation and risk of rupture [5,7,8]. The primary objectives are to prevent ruptures and patient mortality, while considering the risks of invasive therapies [5,8]. Treatment options for HAAs include open surgical repair and endovascular interventions, such as stent graft placement, or coil embolization [7,8]. Recent studies have highlighted the role of minimally invasive techniques such as coil embolization and stent graft placement, as safe and effective alternatives to open surgery [7,8]. Hereby, endovascular approaches have been shown to have success rates of up to 89.7%, presenting safe and effective treatment alternative [7]. In alignment with these findings, we present a case study of a 65-year-old patient with an HAA who was treated using endovascular techniques.

## Case presentation

A 65-year-old male patient was referred to our clinic for interventional radiology, after an incidental HAA was diagnosed during abdominal staging using contrast-enhanced CT-scan, which was performed due to underlying prostate carcinoma. He had undergone a Da Vinci-assisted radical prostatectomy due to the prostate carcinoma. After prostatectomy, the prostate-specific antigen was below the detection limit.

Regarding the HAA, the patient had not experienced any symptoms such as abdominal pain or jaundice. The physical examination did not show any pathologies. The nonsmoking patient had a good general and nutritional status (BMI: 22 kg/m^2^) without known risk factors for HAA, such as vascular disease, pancreatitis or connective tissue disorders. Furthermore, the patient did not have diabetes mellitus, renal disease or coronary artery disease.

Following, we repeated the CT scan to update the imaging and additionally performed MRI scan to further analyze the outline of the aneurysm, showing a saccular aneurysm of 2.6 by 3 cm in diameter. The decision was made to perform an interventional endovascular treatment of the saccular aneurysm under local anesthesia, after an interdisciplinary discussion of the case among vascular surgeons and interventional radiologists.

A 6-French (Fr) introducer sheath was passed through the right femoral artery and into the abdominal aorta, after which an overview contrast angiography was performed using a pigtail catheter (4-Fr). A 4-Fr Cobra catheter and a 5-Fr Sidewinder catheter were then used to replace the pigtail catheter to locate the celiac trunk and common hepatic artery. A 2.4-French microcatheter was left in situ to map the path of the vessels. Following, a Dyna-CT scan and angiogram were performed to confirm correct placement. Two Bentley covered stents 5 mm/24 mm, and 5 mm/20 mm were inserted via a Terumo guidewire to exclude the aneurysm, subsequently balloon dilatation was performed. The final angiogram showed complete exclusion of the aneurysm after stent implantation. After the intervention, the patient was monitored. During the hospital stay, the patient was stable with no complaints. However, the patient’s blood pressure was noticeably high, and a history of uncontrolled hypertension was discovered. However, this did not affect the procedure or the postinterventional state. Treatment with bisoprolol (2.5 mg/day) was then commenced. In addition, dual antiplatelet therapy was started in accordance with current guidelines developed by the American College of Cardiology/American Heart Association and the European Society of Cardiology to minimize thrombotic complications and ensure stent patency. The drugs administered were clopidogrel 75 mg once daily and acetylsalicylic acid (Aspirin) 100 mg once daily for the next 3 months. The patient was then educated about the potential side effects of the dual antiplatelet therapy. After a 3-month period, the patient’s regimen will be altered to monotherapy with aspirin.

**Fig. 1:**
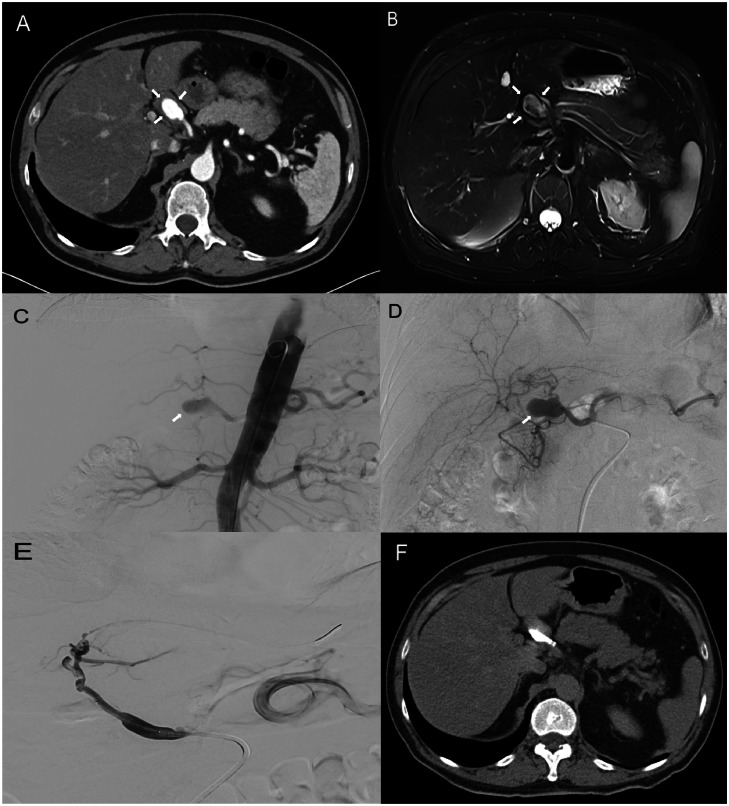
(A and B) Preinterventional contrast enhanced axial CT and axial T2-weighted fat-saturated MRI, show a saccular structure adjacent to the hepatic artery, representing the partially thrombosed saccular aneurysm (white arrows) of the proper hepatic artery with an approximate size of 2.6 × 3 cm. (C) Overview angiography using a pigtail catheter to demonstrate the branches of the abdominal aorta. (D) Selective catheterization of the hepatic artery, hereby the saccular aneurysm bordering the hepatic artery can be seen (white arrow). (E) Shows the vascular state after stent implantation, wherein the aneurysm is no longer perfused. (F) Postinterventional axial CT-scan did not show any complications.

Pre-, intra- and postinterventional images of the patient’s case are shown in Figure 1.

The patient came for a follow-up after 18 weeks and was doing well without abdominal pain or any complications. The liver function tests were checked by the general practitioner and showed normal values. His arterial hypertension was also under control.

## Discussion

HAAs are uncommon vascular abnormalities that may pose a serious risk due to potential rupture, therefore timely diagnosis and intervention is necessary to prevent rupture and possible death [1]. Aneurysms of the common hepatic artery are either considered fusiform, when the whole circumference of the vessel is included, or saccular if only a portion of the vessel is involved [9]. This case demonstrates the effective use of minimally invasive endovascular techniques as a preferred approach, over open surgery, for management of a saccular aneurysm of the proper hepatic artery considering the patient’s baseline function and nutritional status.

Due to the patient’s background of prostate carcinoma and his general health status the most adequate treatment method had to be carefully selected. The patient did not have any abdominal symptoms, underscoring the role of imaging modalities in the identification of asymptomatic aneurysms as incidental findings. Due to the aneurysm’s location, the patient’s stable condition and the benefits of these minimally invasive procedures, the decision was made to undertake an endovascular repair. Compared to open surgery, minimally invasive endovascular techniques have the advantages of reduced recovery time and lower risk of complications [8].

Due to their minimally invasive nature, endovascular techniques, including coil embolization and stent graft placement, have gained popularity in the management of HAAs. They have been shown to lower morbidity rates, and shorten the duration of recovery compared to open surgery [8]. For patients with high surgical risk or comorbidities, these approaches may be especially effective, as shown before in a report published by Vogl et al. [10]. Herein, coil embolization was utilized to successfully treat an HAA in a 83-year old female patient although this patient had undergone several previous vascular procedures, for instance an Octopus operation due to thoracoabdominal aortic aneurysm. However, the long-term effectiveness of endovascular techniques can be limited by complications such as stent thrombosis, incomplete exclusion or reperfusion of the aneurysm (endoleak) [1,7]. For this reason, it is important to have adequate postinterventional monitoring and comprehensive management strategies following endovascular repair, to diagnose potential short- and long-term complications [7]. For these follow-up imaging studies CT-Angiography or regular CT scans should be utilized [10].

Open surgical repair involves aneurysm resection and reconstruction or ligation, and should preferably be utilized for younger, healthier patients due to the better long-term outcomes [2,7]. This approach is particularly advantageous in cases where the aneurysm is large or complex, or is located in a position that makes endovascular intervention challenging. A study by Khan et al. [5] showed that open surgery was the preferred choice for patients with multiple synchronous aneurysms or those requiring vascular reconstruction.

However, Khan et al. [5] demonstrated that complication rates were 32% for patients treated by open surgery and 18% for those treated endovascularly, indicating the benefit of this technique in terms of minimal complications, especially in unstable patients. Furthermore, no significant differences in patency rates between patients treated with open surgery and those treated endovascularly were observed [5]. The 2020 guidelines from the Society of Vascular Surgery conclude that all pseudoaneurysms and all true aneurysms over 2 cm in diameter should be treated, regardless of symptom occurrence and preferably via endovascular surgery [11]. Only large intrahepatic aneurysms should be treated by open surgery to prevent liver necrosis [11].

To conclude, the choice between endovascular and open surgical repair should be made on a case-by-case basis, taking into account the patient’s clinical status, aneurysm morphology, institutional expertise, and available resources. However, endovascular techniques are quick and effective with shorter hospitalization and are likely to continue to gain traction as device technology evolves and should therefore be preferentially chosen, but open surgery remains indispensable for complex cases requiring definitive anatomical correction. Consequently, collaboration between vascular surgeons and interventional radiologists is essential to optimize outcomes for HAA.

## Patient consent

An informed consent was obtained from the patient.
